# The impact of rTMS on patients with dual diagnosis of depressive disorder and substance use disorders

**DOI:** 10.1192/bjo.2021.669

**Published:** 2021-06-18

**Authors:** Wael Foad, Rami Alhawi, Samer Altamimi, Zahid Hussain, Hamdy Moselhy, Mohamed Fayek

**Affiliations:** Erada center for treatment and rehabilitation

## Abstract

**Aims:**

We aim to investigate the effectiveness of repetitive Transcranial Magnetic Stimulation (rTMS) in reducing consumption and craving among patients with Substance Use Disorder (SUD) and comorbid depressive disorder.

**Background:**

Dorsolateral prefrontal cortex (DLPFC) is greatly involved in SUD evolution (1). Research has turned to targeting this brain area with rTMS; a non-invasive brain stimulation technique that modulates cortical excitability by sending pulsatile electromagnetic fields through the skull and into the brain (2). rTMS is an FDA approved and safe treatment option for treatment-resistant depression (TRD) (3).

**Method:**

Fifty-four patients were admitted over six-month period of time (June 2019- December 2019) to the inpatient unit of Erada center for treatment and rehabilitation of SUD in Dubai. All patients who fulfilled ICD-10 diagnoses of Depressive disorder and SUD were screened for further assessment.

Positive drug screen was confirmed through urine analysis. Hospital Anxiety and Depression Scale (HADS) and Brief Substance Craving Scale (BSCS) were applied to all participants. Patients were contracted for 5-times weekly High frequency (10 Hz) rTMS for 4 weeks (total of 20 treatments). Those who managed to complete their contracted TMS sessions were matched for age and sex with similar number of patients who received standard treatment as usual (TAU). Stimulation was as per FDA clearance for rTMS application in TRD.

**Result:**

Eight patients were excluded (previous head trauma). A total of 46 patients had TMS mapping; nine of whom completed 20 sessions.

Opioids was the most commonly used drug in almost 52% of patients (n = 14), followed by amphetamines in almost 30% (n = 8) and Cannabis in 18.5% (n = 5).

Among those who completed 20 rTMS sessions; HADS scores on anxiety and depression fell by 85% and 78% respectively. BSCS score fell by 98%. Relapse rate (defined by positive drug screen) at 3 months was 33%.

For those who completed 10 sessions; there was only 50% reduction on BSCS scores and 66% relapse rate. There were no data available on their HADS scores (only collected at baseline and at completion of 20 sessions).

Those who only had TAU; there were no reduction in their BSCS (average score of 7 at both baseline and after 2 weeks).

**Conclusion:**

Our findings suggest that rTMS may be an effective and safe treatment for both depressive disorder and craving for SUD which is supported by other studies (3,4).

Our study is probably the first of its kind within Middle East population with addiction problems.

